# 

**Published:** 1842-01

**Authors:** 


### CONTENTS OF No. XXV.

OF THE

### British and Foreign Medical Review.

JANUARY, 1842.

---

### Part First.—Analytical and Critical Reviews.

PAGE

Art. I.—1. Ueber die Durchschneidung der Sehnen und Muskeln. Von J. F. Dieffenbach . . . . . . . . 1
On the Division of Tendons and Muscles. By J. F. Dieffenbach.
2. De la Ténotomie sous-cutanée, ou des Opérations qui se pratiquent pour la Guérison des Pieds-bots, du Torticollis, de la Contracture de la Main et des Doigts, des fausses ankyloses angulaires du Genou, du Strabisme, de la Myopie, du Bégaiement, etc. Par le Docteur Ch. Phillips . . . ib.
On Subcutaneous Tenotomy, or the Operations resorted to for the Cure of Club-foot. By Dr. Ch. Phillips.

Art. II.—1. Vergleichende Darstellung der von den Hausthieren auf Menschen übertragbaren Krankheiten. Von Dr. Jacob Levin . . . 28
Comparative History of the Diseases transmissible from Domestic Animals to the Human Race. By Dr. Jacob Levin.
2. De la Morve et du Farcin chez l'Homme. Par M. Rayer . . . ib.
On Glanders and Farcy in the Human Subject. By M. Rayer.

Art. III.—1. Pharmacopœia Castrensis Ruthenica. Auctore Jacobo Wylie, m.d. &c. . . . . . . . . . 54
2. Pharmacopoea Danica, regia auctoritate a Collegio sanitatis regio Hafnensi edita ib.
3. The Pharmacopœia of the Royal College of Physicians of Edinburgh . ib.
4. A Translation of the Pharmacopœia of the Royal College of Physicians of London. By Richard Phillips, f.r.s. . . . . . ib.
5. New Remedies: the method of preparing and administering them; their effects on the healthy and diseased economy, &c. By Robley Dunglison, m.d. . ib.
6. The Elements of Materia Medica; comprehending the Natural History, Preparation, Properties, Composition, Effects, and Uses of Medicines. By Jonathan Pereira, f.r.s. . . . . . . ib.

Art. IV.—Elements of Medicine. Vol. II.—On Morbid Poisons. By Robert Williams, m.d. &c. &c. . . . . . . 89

Art. V.—1. Monographie der Puerperalkrankheiten. Von Theodor Helm, m.d. &c. 100
Monograph on Puerperal Diseases. By Theodore Helm, m.d. &c.
2. Die Krankheiten der Wöchnerinnen nach den in der k. k. Entbindungsanstalt und im allgemeinen Krankenhause zu Prag gemachten Beobachtungen. Von Franz Kiwisch . . . . . . . ib.
The Diseases of Women in Childbed, from Observations made at the Lying-in Institution and General Hospital at Prague. By F. A. Kiwisch, m.d. &c.

Art. VI.—Practical Observations on Injuries of the Head. By Wm. Sharp, f.r.s. 123

Art. VII.—Traité des Névralgies, ou Affections Douloureuses des Nerfs. Par F. L. S. Valleix . . . . . . . 136
A Treatise on Neuralgia, or Painful Affections of the Nerves. By F. L. S. Valleix.

Art. VIII.—Principles of General and Comparative Physiology. By W. B. Carpenter, m.d. . . . . . . . . 160

Art. IX.—The Cyclopædia of Practical Surgery. Conducted by W. B. Costello 168

§

4290

PAGE

ART. X.—1. Rapport sur les Moyens de constater la présence de l'Arsenic dans les Empoisonnemens, &c. Par M. ORFILA . . . . 183
On the Means of detecting Arsenic in Cases of Poisoning, &c. By M. ORFILA.
2. De l'Arsenic, suivi d'une Instruction propre à servir de Guide aux Experts dans les cas d'Empoisonnement, &c. Par MM. DANGER et FLANDIN . . ib.
On Arsenic, with Directions for the Investigation of Cases of Poisoning, &c. By MM. DANGER and FLANDIN.
3. Mémoire sur plusieurs affaires d'Empoisonnement par l'Arsenic, (Mém. de l'Acad. Royale de Médecine, Tome IX.) Par M. ORFILA . . ib.
Memoir on several Trials for poisoning by Arsenic. (From the Memoirs of the Royal Academy of Medicine, Vol. IX.) By M. ORFILA.
4. Recherches Médico-légales et thérapeutiques sur l'Empoisonnement par l'Acide Arsénieux, &c. Recueilliés et redigées par le Docteur BEAUFORT . . ib.
Medico-legal and Therapeutical Researches on Poisoning by Arsenious Acid. By Dr. BEAUFORT.

ART. XI.—Treatise on the Oleum Jecoris Aselli, or Cod Liver Oil, as a therapeutic agent in certain forms of Gout, Rheumatism, and Scrofula; with Cases. By JOHN HUGHES BENNETT, M.D., &c. . . . . . 197

ART. XII.—A New Synopsis, or the Natural Order of Diseases: containing their Definition, Principles, and Treatment, with a New Pathology of Fever and Inflammation. By ROBERT STEVENS, M.R.C.S. &c. . . . 202

ART. XIII.—Pathology founded on the Natural System of Anatomy and Physiology; a Philosophical Sketch, &c. By ALEXANDER WALKER . . . 205

ART. XIV.—Transactions of the Pharmaceutical Society. Edited by JACOB BELL. Nos. I–VI. . . . . . . . 211

### PART SECOND.—Bibliographical Notices.

ART. I.—1. On the Construction and Management of Hospitals for the Insane; with a particular notice of the Institution of Siegburg. By Dr. MAXIMILIAN JACOBI. Translated (from the German) by JOHN KITCHING. With Introductory Observations by SAMUEL TUKE . . . . . 213
2. The Statistics of the Retreat; consisting of a Report and Tables exhibiting the Experience of that Institution for the Insane, from 1796 to 1840 . . ib.
3. The Fifty-ninth Report of the Visiting Justices for the County Lunatic Asylum at Hanwell; and Third Report of the Resident Physician, JOHN CONOLLY, M.D. ib.

ART. II.—Six Letters addressed to the President of Bethlem Hospital, respecting the Management of that Establishment, &c. By PHILANTHROPOS . . 215

ART. III.—Outlines of Comparative Anatomy, presenting a Sketch of the present state of knowledge, and of the progress of discovery in that science; and designed to serve as an Introduction to Animal Physiology, and to the Principles of Classification in Zoology. By ROBERT E. GRANT, M.D. F.R.S.L. & E. F.L.S. &c. 217

ART. IV.—The Physiology of Digestion, considered with relation to the principles of Dietetics. By ANDREW COMBE, M.D. . . . . 219

ART. V.—The Diseases of Children; a Treatise intended for the use of the Student and Junior Practitioner. By AUGUSTUS REES, M.R.C.S. . . . ib.

ART. VI.—The Oration delivered before the Medical Society of London, at their Sixty-eighth Anniversary, March 8, 1841. By W. D. CHOWNE, M.D., &c. . ib.

ART. VII.—A Concise and Practical Treatise on the principal Diseases of the Air-passages, Lungs, and Pleura. By A. CATHERWOOD, M.D. C.M. . . 220

ART. VIII.—Clinical Researches on Auscultation of the Respiratory Organs, and on the First Stage of Phthisis Pulmonalis. By JULES FOURNET . . ib.

ART. IX.—A Practical Treatise on the New Operation for Lateral Curvature of the Spine. By G. B. CHILDS, M.R.C.S., &c. . . . . 221

ART. X.—Popular Lectures on Man, his Structure and Functions, considered with reference to Health, Culture, and Natural Theology. By JOHN WHITE . 222

ART. XI.—A Practical Treatise on the Diseases of Children. By J. STEWART, M.D. ib.

ART. XII.—First Lines of Physiology; designed for the Use of Students of Medicine. By DANIEL OLIVER, M.D. . . . . . . ib.

### PART THIRD.—Selections from the British and Foreign Journals.

### I. THE FOREIGN JOURNALS.

#### ANATOMY AND PHYSIOLOGY.

PAGE
A. W. VOLKMANN on Respiration, with especial regard to Neurological Questions 223
M. LONGET's Experiments on Muscular Irritability and Nervous Action . . 226
on the Nerves of the Larynx, and the Nerve of Willis on Phonation . ib.
Epiglottis, and on the Closure of the Glottis in Deglutition, &c. 227
J. F. SIMON on the Occurrence of Urea in the Blood . . . 228
Dr. FRICKE's Case of Double Vagina, Double Cervix Uteri, and Double Uterus . ib.
Dr. PAPPENHEIM on a Ligament in the Velum Palati . . . ib.
MM. BECQUEREL and BRESCHET on Animal Heat . . . 229
Dr. REMAK on the Production of the Blood-globules . . . ib.
Dr. CRAMER on Absence of the Uterus . . . . . 230

#### PATHOLOGY, PRACTICAL MEDICINE, AND THERAPEUTICS.

Dr. BALLOT's Case, in which an Abscess behind the Œsophagus was mistaken for Œdema of the Glottis, and occasioned death by Asphyxia . . ib.
Dr. J. J. RENAULT on a Form of Diarrhœa which attacks Stokers in Steam-boats . 231
Dr. G. POLLI on the action of certain varieties of Ranunculus . . 232
Professor MULLER on Morbid Parasitic Formation, with Seminal Corpuscles . ib.
M. GIBERT on the New Etiology of Tinea . . . . 233
Dr. BEAU on Rheumatic Dermalgia, or Rheumatism of the Skin . . 234
M. VARLITZ on the Mortality of Leeches after they have been used . . ib.
M. CONSTANTIN JAMES on a Case of Paralysis of the two Facial Nerves . . 235
M. BERARD on Transmission of Glanders from Man to Man . . ib.
M. TROUSSEAU on the Application of Leeches to the Knee in Dysmenorrhœa . 236
M. PELLETAN's Case of Hepato-pleuro-bronchial Fistula . . . ib.

#### SURGERY.

M. AMUSSAT on the Formation of an Artificial Anus . . . 237
M. CIVIALE on Abscesses in the Substance of the Walls of the Bladder . ib.
M. BONNET on the Cure of Myopia and the disposition to fatigue of the Eyes . 238
M. COLSON on Extirpation of the Submaxillary Gland . . . ib.
Dr. DIEFFENBACH on Cure of slight degrees of Squinting without Tenotomy . 239
the Treatment of Old Fractures by Division of Tendons . ib.
Dr. DECONDÉ on Contagious Properties of Gonorrhœal and Blennophthalmic Matter ib.
Dr. JULIUS VOGEL on a rare kind of Stony Concretions in the Skin . . 240
Dr. KEYL's Fatal Stab of the Brain . . . . . ib.
M. MONDIERE's Researches on Puncture of the Bladder . . . 241
Dr. DIEFFENBACH on Paralysis of one side of the Face . . . ib.
M. JULES GUERIN on the Subcutaneous Method of opening Chronic Abscesses . ib.
Drs. LERCHE and KABAT on Curative Influence of Galvanism in Diseases of the Eye 242
Dr. DIEEFENBACH on the Cure of Obliquity of the Nose by Division of the Cartilage ib.
M. TEISSIER on the Effects of Absolute Repose of Joints, without previous Disease 243
M. REYNAUD's Case of Fistula of the Larynx, with Closure of the Tube above it ib.
Dr. DIEFFENBACH on Division of the Muscles of the Face in Chronic Spasms . 244
M. PETEL on a New Mode of Treating Tinea Favosa . . . ib.
M. MALGAIGNE on Strangulated Herniæ . . . . 245
M. MALGAIGNE's Statistical Researches on Dislocations . . . ib.

### MIDWIFERY, AND DISEASES OF WOMEN AND CHILDREN.

PAGE
Dr. L. Ercoliani's Account of a Case of Physometra . . . 246
M. F. Aubry's Account of a General Eruption of Vaccinia at the Hospital Cochin 247
M. Petel on the Performance of Tracheotomy in the last stage of Croup . 248
Dr. Schlesier on Myelitis Infantum . . . . . . ib.
Dr. Schöller on the Employment of the Plug for inducing Premature Labour . 249
Professor P. G. Cederschjöld's Account of an Epidemic of Trismus Neonatorum, which occurred in the Lying-in Hospital at Stockholm in the year 1834 . ib.

### MEDICAL JURISPRUDENCE AND TOXICOLOGY.

Dr. Graff's Case of alleged Childmurder . . . . 250

### ANIMAL CHEMISTRY AND PHARMACY.

MM. Cass and Henry on the State in which Urea exists in the Urine . 253
On the Nutritive Qualities of Gelatine . . . . . ib.
M. Simonin on the Preservation of Ferruginous Pills . . . 254

---

### II. THE BRITISH JOURNALS.

### ANATOMY AND PHYSIOLOGY.

Dr. Craigie on Obliteration of the Aorta beyond the Arch . . . 255
A. Dodd on the Fœtus Viable at Six Months . . . ib.
E. Quekett on the Production of the Ergot of Rye . . . ib.
Dr. Cavet on the Motions of the Heart . . . . . 256

### PATHOLOGY, PRACTICAL MEDICINE, AND THERAPEUTICS.

Dr. Simpson's Antiquarian Notices of Leprosy and Leper Hospitals in Scotland and England . . . . . . . ib.
Dr. Forry on the Connexion of Climate with Diseases of Malarial Origin . 257
Dr. Griffin on the Application of Mathematics to the Science of Medicine . . ib.
Sir William Burnett on Plague not Contagious . . . . 258
Dr. Browne on Hereditary Tendency to Mental Disease . . . 259
Dr. Stanley on Diabetes . . . . . . . ib.
Dr. Hughes Willshire on the Subjectivity of Disease . . . 260
W. Davidson on the Presence of Sulpho-Cyanogen in the Saliva . . ib.
Dr. M. Hall on the Use of Setons, especially in the Treatment of Paraplegia . 261
Dr. Robert Willis on the Dropsy which follows Scarlatina . . ib.
Dr. Williamson on some Diseases in which Albuminous Urine occurs . 262
Dr. Robert Willis on the Signification and Ends of the Portal Circulation . ib.
Dr. Alexander Ure on Hippuric Acid and its Tests . . . 263

### SURGERY.

Mr. Toogood on Deep-seated Abscess of the Breast . . . 264
Mr. Jacob Bell on Mr. Liston's Isinglass Plaster . . . 265
Mr. Stanley's Case of Complete Dislocation of the Fifth Cervical Vertebra, without Fracture . . . . . . . . 266

---

### Part Fourth.—Medical Intelligence.

New Bills of Mortality for London . . . . . . 267
On the Application of the Collegiate System to the Medical Schools of London . 268
On the Treatment of the Insane, by John Conolly, m.d. . . . 274
Books received for Review . . . . . . . 288

